# Young Adults’ Migration to Cities in Sweden: Do Siblings Pave the Way?

**DOI:** 10.1007/s13524-020-00934-z

**Published:** 2020-11-30

**Authors:** Clara H. Mulder, Emma Lundholm, Gunnar Malmberg

**Affiliations:** 1 Population Research Centre, Faculty of Spatial Sciences, University of Groningen, Landleven 1, 9747 AD Groningen, The Netherlands; 2 Centre for Demographic and Ageing Research, Umeå University, Norra Beteendevetarhuset, 4 tr, Umeå Universitet, 901 87 Umeå, Sweden; 3 Department of Geography, Umeå University, Social Science Building, Umeå Universitet, 901 87 Umeå, Sweden

**Keywords:** Internal migration, Young adults, Siblings, Sweden

## Abstract

Young adult internal migration forms a large share of the influx of people into large cities in the developed world. We investigate the role of the residential locations of siblings for young adults’ migration to large cities, using the case of Sweden and its four largest cities: Stockholm, Gothenburg, Malmö/Lund, and Uppsala. We use register data for the full Swedish-born population of young adults aged 18–28 living in Sweden in the years 2007–2013 and multinomial logistic regression analyses of migrating to each of the four cities or migrating elsewhere versus not migrating. Our point of departure is the paving-the-way hypothesis, which posits that young adults who have a sibling living at a migration destination are particularly likely to move to that destination, more so than to other destinations. Additional hypotheses are related to having more than one sibling in the city and to the gender of siblings living at the destination. We find support for the paving-the-way hypothesis and an additional effect for having more than one sibling in the city. Having a sibling of the same gender in a city matters more for moving there than having a sibling of the opposite gender.

**Supplementary Information:**

The online version of this article (10.1007/s13524-020-00934-z) contains supplementary material, which is available to authorized users.

## Introduction

The movement of young people to metropolitan areas forms a large share of internal migration in many high-income countries of today, including Sweden (e.g., Kulu et al. 2018). For young people living outside metropolitan regions, migration to large cities means access to diverse education and labor markets, and may improve the chances of a successful career. In the past few decades, internal migration of young adults and the highly educated has further enhanced the concentration of human capital in the metropolitan areas in Sweden (Eliasson et al. 2019). Thus far, internal migration behavior has mainly been approached using neoclassical economic and human capital perspectives (e.g., Greenwood 1975; Nakosteen and Westerlund 2004). Much of this literature has focused on individuals.

To gain a better understanding of internal migration, it is also important to take the nonresident family into account (Mulder 2018). Failing to do so may lead to a false picture in which migration decisions are erroneously considered as being made by individuals or households searching for economic gains without taking into account the social resources and support that nearby family members may offer. Indeed, evidence shows that nonresident family plays an important role in migration within contemporary high-income societies. For example, several studies have shown that living close to parents (Ermisch and Mulder 2019; Michielin et al. 2008; Mulder and Malmberg 2014; Zorlu 2009), extended family more generally (Spilimbergo and Ubeda 2004), or siblings (Mulder and Malmberg 2014; Zorlu 2009) decreases the likelihood of moving long distances. Other work has shown that family members living elsewhere form an attraction factor for long-distance moves (e.g., Michielin et al. 2008; Pettersson and Malmberg 2009) and intraurban mobility (Hedman 2013; Spring et al. 2017).

Most of the literature on moves toward family has focused on relationships between parents and adult children, and on whether such moves facilitate intergenerational support exchange (e.g., Smits 2010). However, for young adults who still live with their parents or close to them, siblings may in fact be the most important family members to consider. Not only could siblings who migrated first provide valuable support to facilitate migration, but moves toward siblings also indicate that young adult migration runs in families. A finding of strong associations between siblings’ migration and destination choices would indicate an important family impact on how young adults shape their life courses, not only in the sense of spatial mobility but likely also in the sense of educational and labor market outcomes.

In the literature on migrating toward family, siblings have occasionally been considered as additional attraction factors (Michielin et al. 2008; Pettersson and Malmberg 2009) but rarely as the main focus. In one of the few studies to take the location of siblings into account in the location choices of young adults, Berck et al. (2016) found that the presence of siblings was significantly associated with these choices. However, they did not focus specifically on migration but on location choice in general.

Given the possibly important role of siblings and the dearth of empirical evidence on this topic, we present a study on the associations between the presence of siblings in the destination area and young adults’ migration to the core areas of the four largest city regions in Sweden. Sweden is one of the very few countries where rich register data on the entire registered population from the national statistical office are available to researchers. These data contain longitudinal individual-level information about sociodemographic, socioeconomic, and housing characteristics and events, and also on family relations. We use such register data for 2007–2013 and multinomial logistic regression of moving to each of the four largest cities in Sweden (Stockholm, Gothenburg, Malmö/Lund, and Uppsala) or migrating elsewhere versus not migrating (that is, not moving or moving only a short distance).

## Theoretical Framework and Hypotheses

### The City in Young People’s Life Courses

Young adults’ presence in centers of high educational and job opportunities—and thus, particular urban regions—could be beneficial to their later labor market careers. Obviously, getting the best possible education is important for occupational achievement and personal development in general. Migration is frequently required to enroll in such education, particularly for those growing up in areas where educational institutions are not abundant. Eliasson (2006) found that the probability of enrollment in university education in Sweden was associated with access to education and that the enrollment of the less privileged population categories was particularly sensitive to accessibility. Likewise, in contexts where contrasts between urban and less urban areas are large, migration to urban areas is a necessity for many. This is the case in Sweden, where jobs—particularly specialized and well-paying jobs—are concentrated in and around large cities outside the commuting range of large proportions of the population. Fielding (1992) claimed that the metropolitan region of southeast England worked as an “escalator region” facilitating upward social mobility (see also Champion 2012). For the Netherlands, Mulder and van Ham (2005) found that having spent time in the major urban areas was positively related to men’s (but not women’s) occupational achievement later in life. For the United States, Wheeler (2006) demonstrated that men’s wage growth was greater in metropolitan than other areas; and for Sweden, Eliasson and Westerlund (2019) found higher wage premiums from moves up the regional hierarchy.

### The Role of Social Networks in Migration: Why Siblings Would Pave the Way

The importance of social networks to migration has mainly been emphasized by theorists concerned with international migration. According to the migration network theory, the continuation of international migration can be partly explained from facilitating networks of previous migrants in destination areas who provide social capital through kinship, friendship, or community ties (Boyd 1989; Faist 2000; Massey et al. 1993). Although migration network theory has been developed in the context of international migration, social networks are also very likely to be important to internal migration. The social network may in fact be particularly relevant for the migration of young adults (who have not had much time to accumulate resources) to large cities (where housing costs tend to be high and housing is difficult to come by).

Family members tend to be major support providers and important members of people’s social networks (Bengtson 2001; Rossi and Rossi 1990), also in strong welfare states (Dykstra 2009; Sundström et al. 2006). Research from Sweden has revealed how close family relations result in economic support from parents to children (Lennartsson et al. 2010). Even though parents and children tend to have the closest relationships (Bengtson 2001), siblings are often also important network members (Cicirelli 1995; Voorpostel and Blieszner 2008). Eriksen and Gerstel (2002) showed that it was very common for adult siblings to provide some kind of help to each other (see also Voorpostel and van der Lippe 2007).

As Weaver et al. (2003) argued, young adult siblings fulfill specific roles. Among these roles, three seem to be specifically relevant for migration toward siblings. The first is directly offering each other services, such as lending money, allocating other resources, providing help, doing favors, introducing others, and providing care. The second is teaching each other skills and abilities. The third is regulation of behavior through either explicit advice or following each other’s example. In the context of migration to cities, siblings may help arrange housing opportunities or provide shelter, facilitate the search for jobs, and provide all kinds of insider information about the area. Furthermore, they may provide companionship and may introduce their sibling to their own circle of friends. Siblings living in a given city could therefore facilitate migration to that city.

Similarities between siblings in migration behavior may also arise from the way that parents or families shape this behavior. In certain contexts, young adult migration tends to be the outcome of a decision by the parents or a joint decision in the family (Massey 1990; see also Deepak 2005 for migration from Southeast Asia to the United States). Although this pattern may not be the most common in the North European context, the decision to move is obviously shaped in a social context. In some families, migration and getting an education or finding a job in a large city will be valued more than in others. Children from such families will be more likely to be socialized toward migration in general or toward moving to a certain destination in particular. Conversely, in some families, proximity may be particularly valued. For some of these families, this may imply siblings are encouraged or socialized to move to the same destination. Other families may value staying in an area they regard as home. Because we study young adults’ migration from the region in which their parents live, members of such families would be observed to be unlikely to move.

Thus, our main hypothesis is that young adults are more prone to move to a large city if they have a sibling residing there. We refer to this hypothesis as the *paving-the-way hypothesis* because it posits that siblings who migrate first pave the way for those who follow. We regard this hypothesis as supported if we find that those with at least one sibling residing in one of the major cities are more likely to migrate to that particular city than only children and those whose siblings live elsewhere. This increase in likelihood of moving should be found in comparison with not migrating but also with migrating to other cities or elsewhere.

Because having *more than one* sibling at the destination should add to the availability of social capital and could form an additional attraction factor for moving (cf. Pettersson and Malmberg 2009), we expect it to have a positive effect on the likelihood of moving there compared with having only one sibling at the destination. (We use the term *effect* to denote statistical associations, without necessarily implying a causal relation.)

### Gender Differences in Moving Toward Siblings

Gender differences in migration behavior at young adult ages can be understood from gender differences in the timing of higher education entry, labor force entry, partnering, and family formation (Bernard et al. 2016). Because of these differences in the timing of life course events, the age profile of migration tends to peak at a younger age for women than for men. Women are also more likely to move upon partnership formation (Brandén and Haandrikman 2019).

Furthermore, according to Forsberg (1998), the feminization of migration from rural to urban regions was caused by regional variations in the *gender contract* regarding access to labor market opportunities, political influence, and education. She claimed that traditional industrial and agricultural regions provided few alternatives for women apart from low-paid jobs and a traditional role in the family, whereas metropolitan escalator regions offer women access to higher education, better jobs, and influence. For the early twenty-first century, an increased likelihood of migration among women compared with men has been associated with rapid educational expansion among women, for example, in Sweden (Chudnovskaya and Kolk 2017; Johansson 2016) and Germany (Kröhnert and Vollmer 2012).

Because of gender differences in the importance attached to family (Rossi and Rossi 1990), there may also be gender differences in the role of siblings in migration behavior. With regard to the *gender of the sibling*, Eriksen and Gerstel (2002) argued that care work is gendered also among siblings, with sisters providing more help than brothers (see also White and Riedmann 1992). Furthermore, Weaver et al. (2003) hypothesized that pairs of siblings of the same gender would perform more sibling functions than opposite-gender siblings, but they found this only for sister-sister pairs. Accordingly, we would expect having a sister living at the destination to matter more to moving to a particular city than having a brother living there, and having a sibling of the same gender to matter as well, particularly for women. However, location patterns in families in Northern Europe tend to be characterized by patrilocality, with more dispersal among sisters than brothers (Blaauboer et al. 2013; Ghosh et al. 2018). One might therefore alternatively expect that having a brother living at the destination city matters particularly for men.

### Moving Toward Which Siblings: Age, Education, and Student Status

Besides gender, other characteristics of siblings could be associated with the likelihood of moving toward the sibling. For example, Voorpostel et al. (2007) argued that similarity in age is associated with more shared experiences during childhood and could therefore lead to more closeness—and thus support—during adulthood. Alternatively, older siblings could be more likely to be an example for younger siblings. According to White and Riedmann (1992), social support among siblings is lower for those with lower education. Furthermore, a sibling who is enrolled in education can provide specific insider information about student life in the city of destination. We therefore explore whether it matters for the effects of having a sibling at the destination if these siblings are of similar age, older, highly educated, and enrolled in education.

### Alternative Explanations for Sibling Effects on Migration Propensities

We need to consider the possibility that the clustered moves of young adults to a certain city might have other causes than siblings following each other or families promoting moves to that city. For example, young adults may follow their peers rather than their siblings, and peer groups may overlap among siblings. It could also be that certain high schools or teachers promote certain universities, colleges, or occupations located in the city, while siblings tend to go to the same high school. Another possibility is that migration to the same destinations could be a by-product of the young adult pursuing a similar education or career as a sibling—for example, after socialization in the family toward attending particular prestigious universities. Because of gendered socialization, such similarities may also form an alternative explanation of why siblings would be more likely to follow a sibling of the same gender (see also Bras and Neven 2007). It could also be that because of shared upbringing or similarities in genetic dispositions, siblings share unobserved traits that are associated with preferences for migration or for specific locations. Examples of such traits could be an adventurous attitude, a preference for city life, or autonomy with respect to parents. In the current study, we cannot do much to rule out such alternative explanations.

It is also possible that previous migration of siblings is associated with a greater likelihood of any migration rather than with the choice of a particular destination. Young adults may be socialized toward migration in general, and some of them might even deliberately choose to move to a different city than their siblings to avoid sibling competition. If that is the case, a *migration propensity* effect is at work. If the effect of having a sibling in a particular city is larger for moving to the city than for migration to other destinations, we still regard the paving-the-way hypothesis as supported when a migration propensity effect also operates; if the effects are of similar size, we regard the paving-the-way hypothesis as not supported.

### Other Factors Associated With Migration to Urban Areas

It is important to take into account other factors that influence migration of young adults. At the level of the *individual*, age should be accounted for because of the strong age specificity of migration (Bernard et al. 2016). Level of education is known to be strongly associated with the likelihood of migration, and current or prospective students are particularly likely to move (Lundholm 2007). In addition, educational expansion has been associated with longer intergenerational distances (e.g., Chudnovskaya and Kolk 2017). Because individual resources are likely to matter, we account for the individual’s income and unemployment. Ties to partners and children in the household are also known to reduce the likelihood of migrating (e.g., Fischer and Malmberg 2001). Finally, migration history matters: those who live in their area of birth are less likely to migrate than those who live elsewhere (Michaelides 2011).

At the level of the *parental family*, it is important to consider siblings living in the area of origin. These siblings could signify local ties that could keep the young adults from migrating (cf. Mulder and Malmberg 2014), while having other children at home could decrease the amount of resources parents have available to support their child. Furthermore, having siblings living close by could signify that the family values proximity. One of the parents may live at the destination. If this is the case, and a sibling also lives there, an observed move may be directed at least as much toward the parent as toward the sibling. The parents’ migration history should also be taken into account: socialization toward migration to a particular destination city, or toward migration in general, will be more likely when the parents have a history of having lived in the city of destination or elsewhere. Conversely, parents living in their region of birth may indicate a family’s preference for staying in the home region. Parental resources, indicated by their level of education and their income, may help the young adult realize a migration ambition. Parents who do not live together may have fewer opportunities to provide help, but separation of the parents may also lead their children to move away from them (Aquilino 1991).

At the level of the *location of origin*, a greater distance to the large city will be accompanied by a greater barrier to move there (according to the principle of distance decay) and a greater likelihood that a suitable job or educational institution can be found at a location closer by (according to the principle of intervening opportunities). If the location of origin is a densely populated area that offers enough job opportunities and educational institutions, or is a university or college town, the location of origin itself offers intervening opportunities. We would then expect a smaller likelihood of moving to a large city than if it is a smaller settlement. If the local unemployment rate is high, we would expect a greater likelihood of moving out. Finally, we need to take into account the period of the potential move to correct for changes in migration propensities related to, for example, business cycles.

## The Swedish Context

The Swedish context is an interesting case for several reasons. Sweden is a fairly large country with a small number of clearly identifiable centers of high-level jobs and education that are outside the commuting range of many inhabitants. It is not particularly family-oriented (Reher 1998). It is also one of the most egalitarian countries in the world (OECD 2016), with no tuition fees for education and rather generous opportunities for student loans. Hence, young Swedish adults are less dependent on the fortunes of the family than young adults in many other countries. If an impact of the residential location of siblings is found in this country, it is not likely that this impact is idiosyncratic and related to a context in which family is particularly important.

Sweden is characterized by a young age of leaving the parental home (Angelini et al. 2011) and a low share of young adults living with parents (Eurostat 2015). Nevertheless, Swedes tend to enter education at higher ages than the average of OECD countries (OECD 2016), and the age for entering the labor market has risen over time (Uusitalo 2011). Hence, many young Swedes experience a long phase of transition before they become established in the housing and labor markets, with frequent moves during this phase (Kulu et al. 2018). Sweden is also a country with a high level of internal migration—almost as high as in the United States (Bell et al. 2018). Unlike in the United States (Cooke 2018), no recent decline in internal migration has been observed in Sweden (Kulu et al. 2018; Shuttleworth et al. 2018).

More than 50% of the population is concentrated in the three major regions surrounding Stockholm, Gothenburg, and Malmö. These regions also include the four largest cities of Stockholm, Gothenburg, Malmö/Lund, and Uppsala. These cities are major nodes for both internal and international in-migration, with young adults comprising the main inflow. They house the four largest universities in the country (Swedish Higher Education Authority 2018). High-skill jobs are more available in the large cities than elsewhere.

The direct costs of education do not differ between universities in Sweden as they do in, for example, the United States. Yet, social and geographical sorting of students is found (Eliasson 2006), which could be related to high costs of living and housing in the major urban regions. Because of population growth and insufficient housing construction, housing prices have increased substantially in metropolitan Sweden, especially in Stockholm and Gothenburg. Rental housing is hard to access in the Swedish cities (Blind et al. 2016; Granath Hansson 2019). Therefore, many young people moving to the cities depend either on economic support from parents or on contacts to get subtenant contracts or access to housing on the black market.

The capital region of Stockholm is by far the largest population center and the major in-migration region, with the largest supply of higher education and a diverse labor market. The urban core of the region, including the municipality of Stockholm and a number of adjacent municipalities with a distinct urban character, is the most attractive for young in-migrants. Gothenburg and Malmö have traditionally been important centers for industry and trade, but they have increasingly become characterized as having a knowledge-based economy, and higher education has become more important. The core area of the Malmö region includes the large university of Lund. As the fourth-largest city in the country having a large university, Uppsala has a unique role for student migration.

## Data and Methods

### Data Set

For the empirical analyses, we used Swedish register microdata covering all residents registered in the country, provided by Statistics Sweden. The data include annually updated socioeconomic information, residential locations, and place of birth. Importantly, the data also contain links to parents and to siblings (including half-siblings but not stepsiblings) via the parents. We focus on young adults aged 18–28 because in Sweden, these are the ages when most of the increases in distances between parents and siblings take place (Kolk 2017). The population at risk of moving includes those young adults who lived outside three metropolitan local labor market areas (LLMs) as defined by Statistics Sweden (2015)—the Stockholm area (which includes Uppsala), the Gothenburg area, and the Malmö area (which includes Lund)—and who had at least one parent living in the same local labor market area. Young adults without siblings (6% of our study population) are included in the sample.^1^ Of the Swedish-born aged 18–28, 43% meet these criteria. We excluded immigrants (around 8%) because fewer than one-half of them could be linked to a mother in Sweden and level of education was frequently missing for them. We analyzed migration between the end of December of six pairs of years *t*_0_ and *t*_1_: 2007–2008 up to 2012–2013, which are the latest years for which data were available to us.^2^ Observations were treated as censored if a move to a different LLM occurred between *t*_0_ and *t*_1_. We excluded 1.6% of the cases for which level of education was missing for the index person or for both parents. In total, 2,810,636 person-years were included in the analysis, in which 82,292 moves to one of the four cities and 132,642 moves elsewhere were observed.

### Variables

Our migration variable consists of six categories: moved to the core area (city) of Stockholm, Gothenburg, Malmö/Lund, or Uppsala between *t*_0_ and *t*_1_; migrated elsewhere over distances of 50 km or more; or stayed in the current region of residence (reference category). For the dependent variable, we preferred cities over LLMs because the latter are very large, particularly in the metropolitan areas, and do not reflect the daily mobility range of young people who are either students or early in their labor market careers. We also wanted to make sure that the areas were small enough to assume that the potential movers would be likely to be in contact with their siblings. The city of Stockholm was defined as the municipalities that are fully or partly included in the urban area of Stockholm: that is, Solna, Sundbyberg, Sollentuna, Täby, Nacka, Huddinge, Botkyrka, Järfälla, Haninge, Danderyd, Tyresö, and Stockholm. For Gothenburg, we used the municipalities of Gothenburg and Mölndal; for Malmö/Lund, the municipalities of Malmö and Lund; and for Uppsala, the municipality of Uppsala. We grouped Malmö and Lund because of the short distance between these cities (approximately 20 km; 13 minutes by train). For the years for which we had access to data on the dwelling level (2011–2013), we also explored whether the index person moved into the same dwelling as where the sibling lived, but this was rare (2.8% of those who moved to a large city).

With a few exceptions (discussed later), all independent variables were measured at *t*_0._ Our main independent variables are indicators of (1) whether a sibling or more than one sibling lived in each of the four urban centers versus no sibling in each of the urban centers (this reference category includes those without siblings); (2) whether at least one sister lived there, no sibling, or only brothers (reference category); and (3) whether at least one brother lived there, no sibling, or only sisters (reference category). In total, 13.7% of the index persons had at least one sibling living in one of the four cities. For additional analyses, we also used similar indicators of having at least one sibling of similar age living in the cities (between three years younger and three years older), at least one sibling of more than three years older, at least one sibling who had completed postsecondary education, and at least one sibling who was enrolled in education.

Of the other independent variables measured at the level of the *individual*, gender was used as a stratification variable. Age was measured in three categories: 18–21, 22–24, and 25–28. Level of education was categorized as primary, secondary, and postsecondary. The variables enrollment in education and unemployment were derived from information about annual income from student allowances (including student loans) and unemployment benefits. If the index person received any income from these sources during a year, the indicator of interest was coded as 1. This implies that the dummy variable for unemployment should be interpreted as an indicator of having an insecure labor market position. Furthermore, because Swedish young adults frequently migrate to enroll in education, the indicator of enrollment in education was coded as 1 not only if a student allowance was received in *t*_0_ but also if this was the case in *t*_1_. Individual disposable income (in 100,000s of Swedish crowns) was derived from the tax register; the few registered negative incomes were recoded to 0. We used dummy variables to measure whether the index person lived with a parent, was married, and lived with children. Unfortunately, the data do not allow us to identify unmarried cohabitation (which is, in fact, very common in Sweden). The previous migration history was derived from the county of birth and was coded as born in county of residence, born in the county of each of the four cities, or born elsewhere in Sweden. Those born outside Sweden were not included.

At the level of the *family of origin*, a dummy variable indicates whether a sibling lived in the LLM of origin at *t*_0_. Given that at least one parent lived in the LLM of origin at *t*_0_, a categorical variable indicates whether the other parent lived in one of the four cities or elsewhere. The parents’ migration history measures whether at least one parent was born in the county of each of the four cities or, if this was not the case, elsewhere in Sweden or abroad. The variables for parental education and income are based on the same measures as those for the index person. For education, we used the highest known level of the two parents; for income, we added the two incomes. A dummy variable indicates whether the parents do not live together (i.e., they have separated or one is not alive).

At the level of the *LLM of origin* (Sweden has 70 LLMs), the distance to each city was measured in 100 km. We further use indicators of population size in 100,000s at *t*_0_; whether there was a full university or, if not, a university college (i.e., a higher education institute that offers only a limited number of disciplines and does not hand out PhD degrees) in the LLM; and the unemployment rate. Finally, we included dummy variables for the year of observation (*t*_0_). Descriptive statistics for all variables and migration percentages across the categories of the nominal independent variables are presented in Tables A1 and A2 in the online appendix.

### Analytical Strategy

We employed multinomial logistic regression models of moving to each of the four cities or migrating elsewhere in Sweden versus staying. Because of the possible complexity of gender differences, we performed separate analyses for men and women. Because including all sibling indicators in one model would lead to collinearity problems, we present results from three sets of models. The first set includes categorical variables for having one, more than one, or no siblings in each of the four cities, and the control variables. We use these models to test the hypotheses on the effects of having a sibling in the city and having more than one sibling in the city. The second and third set include the variables measuring, respectively, whether a sister or a brother is living in each of the cities, along with the control variables; these models were used to test the hypotheses on the gender of the sibling. The standard errors were corrected for the clustering of index persons in LLMs (the highest-level unit of analysis at which variables were measured). The coefficients for the control variables are shown for only the first set of models; those for the second and third sets were very similar. Next to odds ratios, we also present marginal effects in terms of predicted probabilities for the first set of models in the online appendix. Marginal effects are more easily interpretable to those who are used to thinking in terms of probabilities rather than odds, but for our analyses, they also have an important downside: unlike odds ratios, their magnitude is affected by the size of the city of destination.

We also ran models including indicators of having at least one sibling of similar age living in the cities, at least one older sibling, at least one highly educated sibling, and at least one sibling who was a student. We discuss the results of these models briefly without showing the results.

To check the robustness of our results, we estimated similar models including fewer control variables and models only for students. The results of these models (not shown) were similar to those of the models we present. We also ran separate models for only those who had siblings as well as for those who had only one sibling, two or more siblings, and three or more siblings. Most of the results from these models were also similar; we discuss those that were different briefly without showing the results.

## Results

### Results for Having One or More Siblings in the Cities

The first set of models includes indicators for having one sibling and more than one sibling in the large cities (Table 1 for men and Table 2 for women). A consistent finding for both men and women and for all four cities is that those who have a sibling living in a particular city are considerably more likely to move to that city (odds ratios ranging from 2.36 for women moving to Stockholm to 4.15 for both men and women moving to Uppsala). In terms of marginal effects (see the online appendix, Tables A3 and A4), the predicted annual probability of moving to Stockholm is .012 greater for men, and .016 greater for women if the index person has a sibling there. Given that these are probabilities of moving to only one city in only one year, these effects are quite substantial.

**Table 1:** Multinomial logistic regression of moving (ref. = stayed in region of origin): Odds ratios for men

	Stockholm	Gothenburg	Malmö/Lund	Uppsala	Elsewhere
Siblings in Stockholm (ref. = no)					
One	2.49 ***	1.15 ***	1.10	1.30 ***	1.03 *
More than one	3.07***	1.11	1.37 **	1.40 **	1.05
Siblings in Gothenburg (ref. = no)					
One	1.22 ***	2.83 ***	1.17 **	1.12	1.03
More than one	1.18	3.35 ***	1.06	0.93	0.92
Siblings in Malmö/Lund (ref. = no)					
One	1.13	1.24 ***	3.11 ***	1.43 ***	1.10 ***
More than one	1.34	1.16	4.62 ***	1.02	1.25 **
Siblings in Uppsala (ref. = no)					
One	1.12	1.15	1.60 ***	4.15 ***	1.10 **
More than one	1.03	0.82	0.30 ***	5.50 ***	1.05
Constant	0.00 ***	0.00 ***	0.00 ***	0.00 ***	0.02 ***
*N*	1,510,197				
Log Pseudo-Likelihood	–426,601.00				
Pseudo-*R*^2^	.12				

*Note:* The parameters for the control variables are presented in Table 5.

*p < .05; **p < .01; ***p < .001

**Table 2:** Multinomial logistic regression of moving (ref. = stayed in region of origin): Odds ratios for women

	Stockholm	Gothenburg	Malmö/Lund	Uppsala	Elsewhere
Siblings in Stockholm (ref. = no)					
One	2.36 ***	1.09 *	1.26 ***	1.29 ***	0.99
More than one	2.49 ***	0.91	1.12	1.36 **	0.98
Siblings in Gothenburg (ref. = no)					
One	1.18 ***	2.94 ***	1.05	1.03	1.05 *
More than one	1.33 *	3.52 ***	1.47 **	1.19	1.00
Siblings in Malmö/Lund (ref. = no)					
One	1.20 ***	1.33 ***	3.58 ***	1.21 *	1.05
More than one	1.09	1.43 *	4.77 ***	1.68 *	0.93
Siblings in Uppsala (ref. = no)					
One	1.15 **	1.10	1.21 **	4.15 ***	1.01
More than one	1.27	0.50	2.08 **	4.37 ***	1.07
Constant	0.00 ***	0.00 ***	0.00 ***	0.00 ***	0.02 ***
*N*	1,300,439				
Log Pseudo-Likelihood	–456,649.20				
Pseudo-*R*^2^	.12				

*Note:* The parameters for the control variables are presented in Table 6.

*p < .05; **p < .01; ***p < .001

Those who have more than one sibling living in the city are estimated to be even more likely to make such a move. Furthermore, the odds ratios for having siblings in a certain city are always considerably greater for the likelihood of moving to that particular city than for the likelihood of moving to other cities or elsewhere. Thus, we find convincing support for the paving-the-way hypothesis and also some support for the hypothesis that having more than one sibling in the city of destination would increase the likelihood of moving there even further.

At the same time, there is also evidence of a migration-propensity effect of having one or more siblings in a city. Almost all odds ratios for moving to other cities are between 1.10 and 1.50, and quite a few differ statistically significantly from 1. Most odds ratios for moving elsewhere are close to 1, although for men, there are positive effects of having siblings in Malmö/Lund and Uppsala.

### Results for Having a Sister or Brother Living in the Large Cities

In Tables 3 and 4, the odds ratios are shown for having at least one sister or brother in the city versus having only one or more siblings there of the other gender. Young men have a greater tendency to move in the direction of brothers than sisters: all odds ratios for having a sister in a city and moving to that city are in the range of 0.77 to 0.87, and most *p* values are below .05; for having a brother in a city, all odds ratios are between 1.22 and 1.37. Conversely, young women are more likely to move toward sisters than brothers (with odds ratios of 1.22 to 1.24 for sisters and 0.81 to 0.86 for brothers). Thus, the hypothesis that young adults are particularly likely to move toward a sibling of the same gender is supported, but there are no signs that either sisters or brothers form a greater attraction factor regardless of the gender of the index person. This same-gender effect might be interpreted as support for Weaver et al.’s (2003) hypothesis that pairs of siblings of the same gender would perform more sibling functions than opposite-gender siblings, but it might also be a by-product of the young adult pursuing a similar education or career as a sibling who was socialized in a similar, gendered way and therefore followed a similar migration pathway. For those with only one sibling, the evidence of an additional effect of the gender of the sibling if the sibling lived in the city is somewhat weaker: most of the findings are in the same direction, but the pattern in the odds ratios is more erratic.

**Table 3:** Multinomial logistic regression of moving (ref. = stayed in region of origin), men: Odds ratios from models for having sisters and brothers in the city

	Stockholm		Gothenburg		Malmö/Lund		Uppsala		Elsewhere	
Only Brother(s) in Stockholm (ref.)										
No siblings in Stockholm	0.34 ***		0.77 ***		0.86 *		0.73 ***		0.98	
At least one sister in Stockholm	0.80 ***		0.80 **		0.94		0.92		1.01	
Only Brother(s) in Gothenburg (ref.)										
No siblings in Gothenburg	0.78 ***		0.32 ***		0.84 *		0.83		0.91 ***	
At least one sister in Gothenburg	0.89		0.87 *		0.93		0.83		0.86 ***	
Only Brother(s) in Malmö/Lund (ref.)										
No siblings in Malmö/Lund	0.83 *		0.79 **		0.27 ***		0.65 ***		0.88 ***	
At least one sister in Malmö/Lund	0.91		0.95		0.77 ***		0.83		0.96	
Only Brother(s) in Uppsala (ref.)										
No siblings in Uppsala	0.98		0.94		0.59 ***		0.22 ***		0.89 *	
At least one sister in Uppsala	1.16		1.10		0.80		0.87		0.96	
Constant	0.01 ***		0.01 ***		0.01 ***		0.00 ***		0.03 ***	
*N*	1,510,197									
Log Pseudo-Likelihood	–426,581.10									
Pseudo-*R*^2^	.12									
Only Sister(s) in Stockholm (ref.)										
No siblings in Stockholm	0.45 ***		0.98		0.93		0.80 ***		0.97 *	
At least one brother in Stockholm	1.30 ***		1.27 ***		1.10		1.10		1.00	
Only Sister(s) in Gothenburg (ref.)										
No siblings in Gothenburg	0.87 *		0.39 ***		0.88		0.98		1.05	
At least one brother in Gothenburg	1.12		1.23 ***		1.04		1.16		1.14 ***	
Only Sister(s) in Malmö/Lund (ref.)										
No siblings in Malmö/Lund	0.91		0.83 **		0.36 ***		0.77 *		0.93 *	
At least one brother in Malmö/Lund	1.08		1.03		1.37 ***		1.16		1.06	
Only Sister(s) in Uppsala (ref.)										
No siblings in Uppsala	0.83 **		0.84		0.71 **		0.26 ***		0.94	
At least one brother in Uppsala	0.84 *		0.89		1.17		1.22 *		1.07	
Constant	0.00 ***		0.00 ***		0.00 ***		0.00 ***		0.02 ***	
*N*	1,510,197									
Log Pseudo-Likelihood	–426,567.80									
Pseudo-*R*^2^	.12									

*Note:* The same control variables are included as in the model in Tables 1 and 5.

*p < .05; **p < .01; ***p < .001

**Table 4:** Multinomial logistic regression of moving (ref. = stayed in region of origin), women: Odds ratios from models for having sisters and brothers in the city

	Stockholm	Gothenburg	Malmö/Lund	Uppsala	Elsewhere
Only Brother(s) in Stockholm (ref.)					
No siblings in Stockholm	0.48 ***	0.91	0.88 *	0.77 ***	1.02
At least one sister in Stockholm	1.24 ***	0.95	1.16 *	1.00	1.03
Only Brother(s) in Gothenburg (ref.)					
No siblings in Gothenburg	0.84 **	0.37 ***	0.97	0.87	0.97
At least one sister in Gothenburg	0.99	1.23 **	1.11	0.81	1.02
Only Brother(s) in Malmö/Lund (ref.)					
No siblings in Malmö/Lund	0.78 **	0.69 ***	0.30 ***	0.75 *	0.91 **
At least one sister in Malmö/Lund	0.88	0.85	1.22	0.89	0.90 *
Only Brother(s) in Uppsala (ref.)					
No siblings in Uppsala	0.82 ***	0.94	0.80	0.27 ***	1.03
At least one sister in Uppsala	0.91	0.98	1.01	1.22 *	1.08
Constant	0.01 ***	0.00 ***	0.00 ***	0.00 ***	0.02 ***
*N*	1,300,439				
Log Pseudo-Likelihood	–456,617.50				
Pseudo-*R*^2^	.12				
Only Sister(s) in Stockholm (ref.)					
No siblings in Stockholm	0.38 ***	0.95	0.74 ***	0.78 ***	1.00
At least one brother in Stockholm	0.81 ***	1.05	0.83 *	1.04	0.98
Only Sister(s) in Gothenburg (ref.)					
No siblings in Gothenburg	0.84 **	0.31 ***	0.89	1.05	0.94 *
At least one brother in Gothenburg	1.00	0.84 **	0.93	1.19	0.97
Only Sister(s) in Malmö/Lund (ref.)					
No siblings in Malmö/Lund	0.90 *	0.79 **	0.25 ***	0.85	1.00
At least one brother in Malmö/Lund	1.15	1.12	0.86 *	1.14	1.08
Only Sister(s) in Uppsala (ref.)					
No siblings in Uppsala	0.93	0.93	0.81	0.22 ***	0.98
At least one brother in Uppsala	1.14	0.96	1.04	0.86	0.98
Constant	0.01 ***	0.00 ***	0.01 ***	0.01 ***	0.02 ***
*N*	1,300,439				
Log Pseudo-Likelihood	–456,628.70				
Pseudo-*R*^2^	.12				

*Note:* The same control variables are included as in the model in Tables 2 and 6.

*p < .05; **p < .01; ***p < .001

### Additional Analyses: Age, Education, and Student Status

From the additional analyses (not shown), we found no indication that young adults are more likely to move to a city if an older sibling lived there rather than a sibling who was younger or of similar age. We did find some indications that having a sibling of similar age, a highly educated sibling, or a sibling who is a student in the city of destination is associated with an increased likelihood of moving there compared with having a sibling there without this characteristic. However, no effects of having a sibling of similar age and a highly educated sibling were found in models for those with only one sibling. Possibly, if there is only one sibling, it matters where that sibling lives, but if there are more siblings to choose from, young adults tend to move toward a sibling who is of similar age or highly educated. At the same time, for those who have more siblings, the likelihood that any of them is of similar age or highly educated and lives in the city is greater than for those with only one sibling. The effect of having a sibling of a certain characteristic in the city might therefore be confounded with an effect of having more than one sibling or of having more than one who lives in the city.

### Results for the Other Variables

The age effects differ markedly between men and women, and also between the cities (Tables 5 and 6). Gender differences in age effects are in line with the well-known gendered age patterns of migration (Bernard et al. 2016). The odds ratios for the middle (22–24) and higher (25–28) age categories are considerably larger for moves to Stockholm than for moves to other destinations, for both men and women. This finding is likely related to the differences among the cities in their structures of educational and job opportunities, with Stockholm attracting a larger share of labor migrants (compared with students) than the other cities. In contrast, those in the middle and higher age categories are less likely to move to Uppsala, a typical student city.

**Table 5:** Multinomial logistic regression of moving, men: Odds ratios for control variables

	Stockholm	Gothenburg	Malmö/Lund	Uppsala	Elsewhere
Age Category (ref. = 18–21)					
22–24	1.71 ***	1.25 ***	1.10	1.09 *	1.17 ***
25–28	2.18 ***	1.45 ***	1.25 ***	0.98	1.17 ***
Level of Education (ref. = primary)					
Secondary	1.55 ***	1.19 ***	1.43 ***	1.41 ***	0.96 *
Postsecondary	3.03 ***	1.68 ***	1.83 ***	1.90 ***	0.89 ***
Student	2.60 ***	5.55 ***	9.63 ***	1.64 ***	4.53 ***
Income (SEK 100,000s)	1.09 ***	1.08 ***	1.07 ***	1.08 **	0.93 ***
Unemployed	0.90 *	1.10 *	0.98	0.82 *	1.16 ***
Lives With Parent(s)	2.70 ***	2.36 ***	2.53 ***	2.66 ***	1.55 ***
Married	1.03	0.97	0.82	0.93	1.28 ***
Lives With Child(ren)	0.11 ***	0.14 ***	0.09 ***	0.25 ***	0.39 ***
Migration History (ref. = born in origin)					
Born in Stockholm	1.99 ***	1.08	1.30 **	1.39 ***	1.28 ***
Born in Gothenburg	1.11	1.28 **	1.02	1.13	1.02
Born in Malmö/Lund	1.22	1.14	1.99 ***	1.35	1.42 ***
Born in Uppsala	1.34 **	1.08	1.19	2.17 ***	1.21 *
Born elsewhere	1.25 ***	1.19 ***	1.25 ***	1.14	1.45 ***
Sibling in Area of Origin	0.80 ***	0.81 ***	0.81 ***	0.85 ***	0.78 ***
One Parent Not in Origin (ref. = both in origin)					
In Stockholm	2.47 ***	1.21	1.29	1.05	1.06
In Gothenburg	0.96	2.77 ***	0.51 *	0.77	1.18 *
In Malmö/Lund	1.04	0.58	2.63 ***	1.57	1.12
In Uppsala	1.38	1.44	0.77	3.23 ***	0.94
Elsewhere	1.02	1.06	0.97	0.91	1.59 ***
Parents’ Migration History (ref. = both born in origin)					
At least one born in Stockholm	1.57 ***	1.04	1.18 *	1.43 ***	1.16 ***
At least one born in Gothenburg	1.17 **	1.35 ***	1.33 ***	0.92	1.08 *
At least one born in Malmö/Lund	1.10	1.06	1.54 ***	1.31 **	1.15 ***
At least one born in Uppsala	1.20 **	1.29 **	1.39 **	1.74 ***	1.11 **
None born in four cities, one elsewhere	1.22 ***	1.18 ***	1.24 ***	1.24 ***	1.15 ***
None born in four cities, one abroad	1.30 ***	1.09 *	1.11	1.07	1.07 ***
Parents’ Highest Level of Education (ref. = primary)					
Secondary	1.14 **	1.11	1.39 ***	1.17	1.06 *
Postsecondary	1.69 ***	1.76 ***	2.49 ***	2.16 ***	1.22 ***
Parents’ Income (SEK 100,000s)	1.00 *	1.00 *	1.00 *	1.00 **	1.00
Parents Not Together	1.08 ***	0.95 *	0.94	0.94	1.03 *
Distance to Stockholm (100 km)	0.78	0.75	0.88	1.42	1.00
Distance to Gothenburg (100 km)	1.16 **	0.61 ***	1.35 ***	1.04	0.95
Distance to Malmö/Lund (100 km)	0.98	1.37 ***	0.65 ***	1.10	1.07 *
Distance to Uppsala (100 km)	1.05	1.59 *	1.30	0.53 *	1.03
Population Size (100,000s)	0.85	0.78 *	0.97	0.90	0.81 *
Higher Education in Region (ref. = no)					
College	1.12	1.24	0.92	0.96	0.87
University	1.17	1.18	1.11	0.88	0.87
Unemployment Rate	1.01	1.00	0.97 *	1.01	1.02 *
Year (ref. = 2007)					
2008	0.96	0.93	0.87 **	0.99	1.09
2009	0.96	0.85 ***	0.86 **	0.90	0.98
2010	0.89 *	0.84 ***	0.69 ***	0.86	1.06
2011	0.86	0.78 *	0.59 ***	0.95	1.07
2012	0.86	0.80 **	0.61 ***	0.82	1.10

*Note:* Parameters for main independent variables are in Table 3.

*p < .05; **p < .01; ***p < .001

**Table 6:** Multinomial logistic regression of moving, women: Odds ratios for control variables

	Stockholm	Gothenburg	Malmö/Lund	Uppsala	Elsewhere
Age Category (ref. = 18–21)					
22–24	1.61 ***	1.22 ***	1.18 ***	0.86 **	1.10 ***
25–28	1.92 ***	1.25 ***	1.19 **	0.66 ***	1.09 **
Level of Education (ref. = primary)					
Secondary	1.79 ***	1.55 ***	1.50 ***	1.29 ***	1.05 *
Postsecondary	2.52 ***	1.68 ***	1.63 ***	1.61 ***	0.83 ***
Student	1.86 ***	3.35 ***	6.34 ***	10.34 ***	3.28 ***
Income (SEK 100,000s)	1.21 ***	1.14 ***	1.01	1.11 **	0.91 ***
Unemployed	0.93 *	1.03	0.99	0.93	1.10 ***
Lives With Parent(s)	4.08 ***	3.43 ***	4.05 ***	4.24 ***	2.20 ***
Married	0.84 *	0.65 ***	0.84	1.02	1.05
Lives With Child(ren)	0.15 ***	0.15 ***	0.15 ***	0.28 ***	0.38 ***
Migration History (ref. = born in origin)					
Born in Stockholm	1.73 ***	1.04	1.32 ***	1.22 *	1.17 ***
Born in Gothenburg	1.12 *	1.11	1.10	1.03	0.96
Born in Malmö/Lund	1.07	1.20	1.83 ***	1.17	1.19 *
Born in Uppsala	1.12	1.21	1.03	1.98 ***	1.05
Born elsewhere	1.19 ***	1.18 ***	1.22 ***	1.20 ***	1.37 ***
Sibling in Area of Origin	0.83 ***	0.81 ***	0.82 ***	0.81 ***	0.79 ***
One Parent Not in Origin (ref. = both in origin)					
In Stockholm	1.93 ***	0.82	0.98	1.08	0.97
In Gothenburg	0.98	2.48 ***	0.96	1.06	1.24
In Malmö/Lund	1.02	0.98	1.45 **	1.38	0.96 *
In Uppsala	0.76	0.58	0.78	3.38 ***	1.08
Elsewhere	1.03	0.97	0.89 *	0.98	1.53
Parents’ Migration History (ref. = both born in origin)					
At least one born in Stockholm	1.52 ***	1.19 ***	1.23 ***	1.32 ***	1.12
At least one born in Gothenburg	1.00	1.36 ***	1.18 *	1.08	1.07 ***
At least one born in Malmö/Lund	1.03	1.16 *	1.54 ***	1.27 *	1.09 *
At least one born in Uppsala	1.27 ***	1.22 **	1.43 ***	1.61 ***	1.12
None born in four cities, one elsewhere	1.19 ***	1.21 ***	1.19 ***	1.24 ***	1.13 *
None born in four cities, one abroad	1.30 ***	1.17 ***	1.20 ***	1.03	1.06 ***
Parents’ Highest Level of Education (ref. = primary)					
Secondary	1.19 **	1.20 ***	1.20 *	1.35 **	1.08
Postsecondary	1.76 ***	1.84 ***	2.16 ***	2.61 ***	1.20
Parents’ Income (SEK 100,000s)	1.00 *	1.00 **	1.01 ***	1.01 ***	1.00 ***
Parents Not Together	1.18 ***	1.05 *	1.03	0.94 *	1.05
Distance to Stockholm (100 km)	0.89	0.70	0.84	1.28	0.97 *
Distance to Gothenburg (100 km)	1.17 ***	0.63 ***	1.31 ***	1.12 *	0.93
Distance to Malmö/Lund (100 km)	0.97	1.36 ***	0.68 ***	1.05	1.09
Distance to Uppsala (100 km)	0.94	1.73 **	1.35	0.60 **	1.08
Population Size (100,000s)	0.89	0.87	1.00	0.90	0.79
Higher Education in Region (ref. = no)					
College	1.06	1.10	0.91	0.95	0.90
University	1.13	1.10	1.13	0.93	0.91
Unemployment Rate	1.01	0.99	0.98	1.00	1.02
Year (ref. = 2007)					
2008	1.04	0.88 *	0.92	0.92	1.04
2009	0.98	0.89 ***	0.88 **	0.83 **	0.96
2010	0.93	0.82 ***	0.77 ***	0.81 *	0.97
2011	0.91	0.75 ***	0.69 ***	0.87	1.02
2012	0.88 *	0.75 ***	0.65 ***	0.80	1.04

*Note:* Parameters for main independent variables are in Table 4.

*p < .05; **p < .01; ***p < .001

Higher completed levels of education of the young adults are associated with a greater likelihood of moving to the cities, but no such effects are found for moving elsewhere. Stark positive effects of being a student are found for moves to Gothenburg, Malmö/Lund, and particularly Uppsala, but somewhat less so for Stockholm and for migrating elsewhere. This difference between Stockholm and the other cities may be related to the different functions of the four cities. Stockholm is the country’s major center of economic activity. Income is positively associated with the likelihood of moving to the cities (except for women moving to Malmö/Lund) but negatively associated with the likelihood of migrating elsewhere. For unemployment, there are no large effects, and the findings are mixed.

Those who live with their parents are more likely to move to a city or migrate elsewhere than those who have established a household of their own in the same labor market area as their parents. This is understandable because education and work elsewhere are major occasions for leaving the parental home. As one would expect, those with children are less likely to move to any destination than those without. Women’s moves to Stockholm and Gothenburg are less likely for the married than the unmarried, but unmarried cohabitation is very common in Sweden and not recorded in the data.

Except for women moving to Gothenburg, we find positive effects of having been born in a county containing one of the large cities on the likelihood of moving there in young adulthood. In various cases, there is also a positive but mostly smaller effect on migrating to other destinations. In most cases, having been born in any other labor market area than the current one or the city of interest is also associated with a greater likelihood of moving to the city and elsewhere.

As expected, we find statistically significant negative effects of having a sibling living in the LLM area of origin (odds ratios around 0.8; in most of the models for only those with siblings, the odds ratios were somewhat lower). These effects are found for both men and women and for the likelihood of moving to any destination.

Naturally, having a parent living in the city is associated with a strongly increased likelihood of moving there. Similarly, if a parent was born in the city, there is an increased likelihood of moving there, and in many cases also an increased likelihood of migrating to other destinations. An increased likelihood of moving to the city and of migrating to other destinations is also found if a parent was born in a different labor market area in Sweden. These findings provide another indication that location choice and migration run in families. Having an immigrant parent also has some positive effects on moving to some of the cities, particularly to Stockholm. This finding could be indicative of the diverse labor market in Stockholm and a large immigrant population, which may act as a magnet for those with immigrant backgrounds.

With every additional 100-km distance to Gothenburg, young adults are estimated to be 0.63 times as likely to migrate to that city. Yet, the associations are weaker and nonsignificant for Stockholm. This weaker effect could be another indication of Stockholm’s specific function. Conversely, quite a few positive effects are found of distance to the city on migrating to other destinations, likely indicating the existence of intervening opportunities closer by. We see some indications of a decrease in migration propensities over time, particularly to Gothenburg and Malmö/Lund.

## Conclusions and Discussion

We examined the role that siblings play in young adults’ moves to the largest cities in Sweden (Stockholm, Gothenburg, Malmö/Lund, and Uppsala) or elsewhere versus not migrating. Using register data for the entire population of Swedish-born individuals aged 18–28, we found support for the paving-the-way hypothesis, stating that young adults would be more likely to move to a large city if they had a sibling living in that particular city. This increase in likelihood of moving was found in comparison with both not migrating and migrating to other cities or elsewhere. This latter finding indicates that the sibling effects were indeed specific to destination cities rather than just an indication of increased propensities to migrate to any destination. Yet, indications of increased migration propensities to other destinations were also found. We found the expected additional effect of having more than one sibling in the city. We also found that young adults were particularly likely to move toward a sibling living in a large city if the sibling was the same gender. However, in contrast with theories on gendered care work, we did not find a greater effect of having a sister than a brother in a destination city.

Our findings clearly indicate that migration to large cities runs in families. They are therefore in line with the idea that, as Massey et al. (1993) argued for international migration and Mulder (2018) argued for internal migration, the family is an important context for migration. The findings are also in line with the idea that siblings could be valuable sources of social capital by providing an example, care work, help, information, and/or company. Given what is known about the important role siblings play in social networks and support exchange, it is likely that the sibling effects found in our models are indeed at least partly related to such interactions between the siblings. The fact that effects were greater for migration to the city where the sibling lived than for migration to the other three cities and other destinations also suggests that this is the case.

Our data, however, do not contain information about social interactions. We therefore cannot rule out that the findings are related to one of the alternative explanations of the sibling effects. First, the effects could be at least partly spurious by-products of influence exerted by friends, classmates, other peers, or teachers. However, given recent findings for Sweden indicating that more than one-half of survey respondents who moved toward nonresident family members actually mentioned family as a reason for moving (Gillespie and Mulder 2020), we think it is unlikely that all or most moves toward siblings are related only to such by-product effects. Future research might be able to explore the by-product option further by including register data on residence at the destination of people who were previously enrolled in the same high school or who used to be neighbors (assuming that the young adults would know these people). It may also be possible to employ data on whether siblings attend the same school after the move or have the same occupation in order to find out whether such similarities play a part in siblings following each other.

Second, the sibling effects could be caused by unobserved traits that are shared among siblings, either because of shared socialization or because siblings are genetically predisposed toward similar personality types or preferences. It is hard to see which research designs could tackle this issue. Asking young adults about their motives for moving and choosing a specific destination may help somewhat, but closed-ended questions about motives for moving in existing surveys do not provide the detail needed to uncover whether siblings played a role, and responses to open-ended questions may also be unspecific.

Whatever the causal mechanisms may be, the finding that migration and location choice run in families has important implications. This finding suggests that access to centers of educational opportunities and specialized jobs is partly determined by the family young adults are born into rather than by their talents. Migration toward siblings will also lead to a greater geographical concentration of kinship networks and could lead to greater access to such networks later in life—particularly given Kolk’s (2017) observation for Sweden that distances to siblings do not decrease much after age 28.

It would be of interest to know whether siblings who paved the way have an impact on young adults’ labor market, social, and housing outcomes. We may expect movers who can rely on the social capital of siblings to be more successful in their housing and labor market careers and more likely to become socially integrated in their urban destination. Hence, they may be less likely to lack adequate housing, become unemployed, drop out of higher education, or move back home with or closer to parents. Conversely, if the destination of migration is strongly influenced by siblings’ place of residence but nearby siblings have a marginal effect on how young adults fare socially and economically, perhaps those who move close to siblings would forgo destinations, educations, and career alternatives that might have been better for their future labor market careers.

It would be helpful to replicate our study in contexts other than Sweden, such as in other large countries with high levels of migration, in more familistic societies, and in different welfare regimes, where the need for relying on family might be greater than in Sweden. However, performing such a replication requires data on residential locations of siblings and on migration to rather small areas. This requirement is likely prohibitive in contexts where microdata for entire populations are lacking. In that respect, Sweden is among the very few countries for which adequate data are available.

## Electronic supplementary material

ESM 1 (PDF 638 kb)

## Data Availability

The data are available only at the data lab of CEDAR, Umeå University, in the context of the research program on Ageing and Living Conditions.

## Footnotes

1 The percentage of young adults without siblings may seem low, but it is a percentage of children, not mothers or families.

2 These were the years for which the data had been released by Statistics Sweden and purchased by the institution where we carried out the research.
