# Supplementary material for: Young Adults’ Migration to Cities in Sweden: Do Siblings Pave the Way?
Source: Demography. 2020 Nov 30;57(6):2221–44. doi: 10.1007/s13524-020-00934-z (PMC7732800; doi:10.1007/s13524-020-00934-z)
Supplement: Supplementary file 1 — (PDF 638 kb) [file 13524_2020_934_MOESM1_ESM.pdf]

Online Appendix

Table A1: Descriptive statistics, men (N = 1,510,197)

	% in sam-ple/ *mean*	*std dev*	% moved to: Stock-holm	Gothen-burg	Malmö/ Lund	Upp-sala	Else-where
Whether moved: no	93.29						
To Stockholm	0.98						
To Gothenburg	0.74						
To Malmö/Lund	0.41						
To Uppsala	0.35						
Elsewhere	4.22						
Siblings in Stockholm: no	93.74		0.81	0.73	0.40	0.33	4.16
one	5.51		3.20	0.95	0.54	0.64	5.01
more than one	0.75		5.84	0.94	0.74	0.82	5.86
Sister in Stockholm: brother(s) only	2.57		3.93	1.12	0.62	0.70	5.04
Yes	3.76		3.21	0.84	0.53	0.64	5.15
Brother in Stockholm: sister(s) only	3.31		2.92	0.83	0.51	0.62	5.07
Yes	3.02		4.10	1.10	0.62	0.71	5.17
Sibling same age in Stockholm: older/younger only	4.34		3.12	0.94	0.57	0.69	5.01
Yes	1.95		4.39	0.99	0.57	0.60	5.34
Older sibling in Stockholm: younger/same age only	2.16		4.10	0.97	0.54	0.54	5.12
Yes	4.19		3.18	0.95	0.58	0.73	5.11
Highly educated sibling in Stockholm: less educated only	2.59		3.12	0.67	0.33	0.37	4.73
Yes	3.72		3.79	1.16	0.73	0.87	5.40
Student-sibling in Stockholm: non-student only	4.93		3.26	0.85	0.52	0.63	4.94
Yes	1.34		4.43	1.32	0.76	0.81	5.75
Siblings in Gothenburg: no	96.04		0.97	0.63	0.40	0.34	4.19
one	3.60		1.33	3.22	0.65	0.42	4.86
more than one	0.36		1.22	5.84	0.57	0.32	4.80
Sister in Gothenburg: brother(s) only	1.74		1.42	3.76	0.70	0.47	5.27
Yes	2.22		1.25	3.21	0.60	0.36	4.53
Brother in Gothenburg: sister(s) only	2.05		1.26	2.94	0.61	0.37	4.51
Yes	1.91		1.40	4.00	0.68	0.45	5.22
Sibling same age in Gothenburg: older/younger only	2.42		1.24	3.04	0.62	0.36	4.75
Yes	1.54		1.46	4.10	0.68	0.49	5.01
Older sibling in Gothenburg: younger/same age only	1.74		1.48	3.69	0.63	0.45	4.94
Yes	2.22		1.20	3.27	0.65	0.38	4.78
Highly educated sibling in Gothenburg: less educated only	1.58		1.00	2.73	0.33	0.24	4.35
Yes	2.38		1.54	3.94	0.85	0.52	5.19
Student-sibling in Gothenburg: non-student only	2.60		1.25	2.95	0.49	0.29	4.51
Yes	1.36		1.46	4.42	0.94	0.65	5.51
Siblings in Malmö/Lund: no	98.02		0.97	0.73	0.35	0.34	4.19
one	1.83		1.44	1.38	3.05	0.56	5.44
more than one	0.15		1.69	1.17	6.51	0.39	6.64
Sister in Malmö/Lund: brother(s) only	0.87		1.55	1.41	3.79	0.63	5.70
Yes	1.12		1.38	1.33	2.95	0.47	5.40
Brother in Malmö/Lund: sister(s) only	1.05		1.39	1.34	2.71	0.48	5.33

Yes	0.94		1.54	1.39	3.99	0.62	5.77
Sibling same age in Malmö/Lund: older/younger only	1.14		1.28	1.27	2.87	0.52	5.34
Yes	0.84		1.69	1.49	3.92	0.58	5.80
Older sibling in Malmö/Lund: younger/same age only	0.94		1.65	1.45	3.50	0.56	5.58
Yes	1.04		1.28	1.28	3.15	0.53	5.49
Highly educated sibling in Malmö/Lund: less educated only	0.56		1.01	0.79	2.17	0.26	4.71
Yes	1.42		1.63	1.59	3.77	0.66	5.86
Student-sibling in Malmö/Lund: non-student only	1.08		1.32	1.09	2.81	0.44	5.21
Yes	0.90		1.62	1.69	3.92	0.67	5.92
Siblings in Uppsala: no	98.52		0.97	0.74	0.41	0.31	4.19
one	1.38		1.85	1.04	0.76	2.86	6.11
more than one	0.10		1.74	0.71	0.13	5.54	6.06
Sister in Uppsala: brother(s) only	0.66		1.75	0.96	0.81	3.41	6.25
Yes	0.82		1.91	1.05	0.64	2.76	5.99
Brother in Uppsala: sister(s) only	0.78		1.94	1.08	0.67	2.58	5.95
Yes	0.71		1.74	0.94	0.77	3.56	6.28
Sibling same age in Uppsala: older/younger only	0.83		1.69	0.86	0.64	2.40	5.74
Yes	0.65		2.04	1.21	0.81	3.89	6.58
Older sibling in Uppsala: younger/same age only	0.75		2.11	1.16	0.82	3.41	6.23
Yes	0.73		1.57	0.86	0.61	2.68	5.98
Highly educated sibling in Uppsala: less educated only	0.40		1.27	0.68	0.41	2.23	5.26
Yes	1.08		2.05	1.14	0.83	3.35	6.42
Student-sibling in Uppsala: non-student only	0.71		1.64	0.66	0.42	2.11	5.22
Yes	0.78		2.03	1.34	0.98	3.91	6.92
Age category: 18-21	45.77		0.73	0.76	0.47	0.41	4.91
22-24	26.07		1.23	0.84	0.45	0.39	4.31
25-28	28.16		1.16	0.62	0.29	0.20	3.02
Level of education: primary	24.76		0.48	0.46	0.21	0.18	4.06
Secondary	88.61		0.83	0.65	0.37	0.31	3.98
Post-secondary	11.39		2.93	1.85	1.10	0.90	5.95
Student	21.35		2.27	2.25	1.51	1.33	10.58
Income (SEK 100,000s)	*1.37*	*1.05*					
Unemployed	7.01		0.79	0.57	0.24	0.17	3.92
Lives with parent(s)	56.78		1.27	1.02	0.60	0.51	5.44
Married	2.14		0.41	0.24	0.08	0.07	2.17
Lives with child(ren)	6.28		0.08	0.05	0.01	0.02	0.99
Migration history: both born in origin	86.63		0.88	0.65	0.38	0.32	3.93
Born in Stockholm	2.73		3.44	0.76	0.55	0.73	6.40
Born in Gothenburg	3.02		0.68	2.77	0.37	0.21	4.23
Born in Malmö/Lund	0.66		1.22	1.01	3.14	0.41	6.90
Born in Uppsala	0.67		1.87	0.77	0.46	2.09	5.71
Born elsewhere	6.28		1.31	1.00	0.61	0.42	6.82
Sibling in area of origin	82.65		0.86	0.66	0.37	0.31	3.91
One parent not in origin: both in origin	89.94		0.94	0.73	0.42	0.35	3.99
In Stockholm	1.01		5.41	0.70	0.47	0.53	5.08
In Gothenburg	0.48		0.82	4.85	0.20	0.18	4.67
In Malmö/Lund	0.17		1.15	0.42	4.64	0.50	5.44

In Uppsala	0.13		2.10	0.73	0.21	4.62	4.46
Elsewhere	8.28		0.93	0.62	0.31	0.24	6.61
Parents' migration history: both born in origin	46.55		0.72	0.53	0.28	0.25	3.59
At least one born in Stockholm	4.83		2.19	0.68	0.48	0.61	5.30
At least one born in Gothenburg	5.26		0.77	2.17	0.46	0.21	4.30
At least one born in Malmö/Lund	2.71		0.96	0.89	1.66	0.35	4.95
At least one born in Uppsala	1.66		1.45	0.84	0.48	1.18	4.98
None born in 4 cities, one elsewhere	28.80		1.15	0.80	0.49	0.43	4.82
None born in 4 cities, one abroad	10.18		1.18	0.76	0.42	0.34	4.53
Parents' highest level of education: primary	6.19		0.54	0.39	0.14	0.10	2.86
Secondary	54.82		0.65	0.47	0.22	0.17	3.54
Post-secondary	38.99		1.52	1.18	0.73	0.63	5.40
Parents' income (SEK 100,000s)	*4.98*	*5.20*					
Parents not together	42.62		0.96	0.62	0.32	0.27	4.30
Distance to Stockholm (100 km)	*3.12*	*1.66*					
Distance to Gothenburg (100 km)	*3.53*	*2.62*					
Distance to Malmö/Lund (100 km)	*4.82*	*3.04*					
Distance to Uppsala (100 km)	*3.06*	*1.59*					
Population size (100,000s)	*1.42*	*0.71*					
Higher education in region (ref. no)	26.65		0.98	0.63	0.32	0.36	5.55
College	41.34		0.92	0.94	0.54	0.33	3.57
University	32.01		1.07	0.57	0.33	0.35	3.95
Unemployment rate	*1.07*	*3.47*					
Year: 2007	15.74		1.05	0.79	0.46	0.36	4.12
2008	16.18		0.99	0.77	0.45	0.36	4.30
2009	16.60		1.01	0.73	0.44	0.35	4.26
2010	16.97		0.97	0.74	0.39	0.34	4.40
2011	17.18		0.93	0.69	0.37	0.36	4.06
2012	17.33		0.95	0.72	0.38	0.31	4.17

Table A2: Descriptive statistics, women (N = 1,300,439)

	% in sam-ple/ *mean*	*std dev*	% moved to: Stock-holm	Gothen-burg	Malmö/ Lund	Upp-sala	Else-where
Whether moved: no	91.26						
To Stockholm	1.39						
To Gothenburg	1.00						
To Malmö/Lund	0.58						
To Uppsala	0.49						
Elsewhere	5.30						
Siblings in Stockholm: no	94.04		1.20	0.98	0.56	0.45	5.26
one	5.23		4.21	1.18	0.82	0.85	5.78
more than one	0.74		6.45	0.99	0.84	1.13	6.44
Sister in Stockholm: brother(s) only	2.54		3.96	1.21	0.76	0.89	5.76
Yes	3.51		4.84	1.12	0.86	0.88	5.93
Brother in Stockholm: sister(s) only	3.05		4.67	1.12	0.87	0.85	5.85
Yes	2.99		4.26	1.19	0.76	0.92	5.85
Sibling same age in Stockholm: old-er/younger only	4.22		4.09	1.16	0.78	0.91	5.95
Yes	1.78		5.42	1.16	0.90	0.81	5.63
Older sibling in Stockholm: young-er/same age only	2.01		5.02	1.05	0.86	0.72	5.37
Yes	4.05		4.19	1.21	0.80	0.96	6.11
Highly educated sibling in Stockholm: less educated only	2.52		3.82	0.92	0.56	0.50	5.37
Yes	3.50		4.95	1.33	1.00	1.16	6.21
Student-sibling in Stockholm: non-student only	4.71		4.26	1.08	0.73	0.80	5.73
Yes	1.28		5.28	1.45	1.16	1.20	6.34
Siblings in Gothenburg: no	96.20		1.38	0.86	0.57	0.47	5.28
one	3.46		1.78	4.07	0.79	0.53	5.88
more than one	0.35		1.78	6.56	1.08	0.51	5.81
Sister in Gothenburg: brother(s) only	1.77		1.81	3.85	0.78	0.61	5.86
Yes	2.04		1.75	4.68	0.84	0.46	5.89
Brother in Gothenburg: sister(s) only	1.86		1.77	4.49	0.83	0.47	5.88
Yes	1.94		1.79	4.10	0.80	0.59	5.87
Sibling same age in Gothenburg: old-er/younger only	2.35		1.59	3.86	0.76	0.48	5.85
Yes	1.45		2.09	5.00	0.90	0.61	5.92
Older sibling in Gothenburg: young-er/same age only	1.63		1.93	4.44	0.83	0.53	5.66
Yes	2.17		1.66	4.18	0.81	0.53	6.04
Highly educated sibling in Gothenburg: less educated only	1.52		1.30	3.43	0.51	0.28	5.27
Yes	2.29		2.10	4.87	1.02	0.70	6.28
Student-sibling in Gothenburg: non-student only	2.49		1.65	3.68	0.66	0.35	5.37
Yes	1.32		2.01	5.46	1.10	0.88	6.83
Siblings in Malmö/Lund: no	98.10		1.38	0.98	0.50	0.47	5.28
one	1.76		1.96	1.85	4.34	0.62	6.09
more than one	0.15		1.85	2.01	8.55	0.90	6.02
Sister in Malmö/Lund: brother(s) only	0.85		2.10	2.06	4.06	0.67	6.39
Yes	1.05		1.84	1.70	5.16	0.61	5.83
Brother in Malmö/Lund: sister(s) only	0.98		1.82	1.73	4.93	0.60	5.82

Yes	0.92		2.10	2.00	4.38	0.68	6.36
Sibling same age in Malmö/Lund: older/younger only	1.13		1.69	1.65	4.06	0.54	5.79
Yes	0.77		2.35	2.18	5.55	0.78	6.52
Older sibling in Malmö/Lund: younger/same age only	0.86		2.33	1.99	4.78	0.75	6.26
Yes	1.05		1.65	1.76	4.57	0.54	5.93
Highly educated sibling in Malmö/Lund: less educated only	0.55		1.61	1.31	2.88	0.40	5.45
Yes	1.35		2.10	2.09	5.39	0.73	6.34
Student-sibling in Malmö/Lund: non-student only	1.04		1.75	1.58	3.54	0.37	5.33
Yes	0.86		2.20	2.21	6.01	0.96	6.99
Siblings in Uppsala: no	98.55		1.38	0.99	0.57	0.43	5.28
one	1.35		2.51	1.28	0.81	3.64	6.61
more than one	0.10		2.85	0.55	1.19	6.01	7.51
Sister in Uppsala: brother(s) only	0.67		2.72	1.24	0.81	3.44	6.33
Yes	0.78		2.38	1.22	0.86	4.10	6.95
Brother in Uppsala: sister(s) only	0.74		2.34	1.26	0.85	3.92	6.79
Yes	0.71		2.73	1.19	0.82	3.67	6.53
Sibling same age in Uppsala: older/younger only	0.80		2.21	1.08	0.76	3.01	6.25
Yes	0.64		2.94	1.41	0.93	4.78	7.19
Older sibling in Uppsala: younger/same age only	0.73		2.78	1.34	0.86	4.12	6.93
Yes	0.71		2.27	1.11	0.82	3.46	6.39
Highly educated sibling in Uppsala: less educated only	0.39		2.14	0.57	0.59	2.58	6.00
Yes	1.06		2.68	1.47	0.93	4.24	6.91
Student-sibling in Uppsala: non-student only	0.68		2.40	1.00	0.59	2.50	5.51
Yes	0.77		2.65	1.42	1.06	4.93	7.68
Age category: 18-21	48.06		1.25	1.13	0.70	0.64	6.74
22-24	24.99		1.74	1.12	0.64	0.48	5.02
25-28	26.95		1.34	0.63	0.30	0.18	2.98
Level of education: primary	23.47		0.71	0.59	0.33	0.30	5.37
Secondary	57.01		1.31	1.01	0.57	0.47	5.39
Post-secondary	19.52		2.47	1.44	0.89	0.72	4.95
Student	32.98		2.34	2.03	1.42	1.25	9.66
Income (SEK 100,000s)	*1.13*	*0.79*					
Unemployed	7.16		1.01	0.61	0.27	0.19	3.90
Lives with parent(s)	48.70		2.14	1.60	1.00	0.83	7.92
Married	4.87		0.38	0.17	0.09	0.07	1.95
Lives with child(ren)	15.73		0.13	0.07	0.03	0.03	1.29
Migration history: both born in origin	86.10		1.29	0.90	0.53	0.45	5.05
Born in Stockholm	2.78		4.07	0.94	0.76	0.84	6.85
Born in Gothenburg	3.05		0.96	3.04	0.55	0.30	5.15
Born in Malmö/Lund	0.70		1.41	1.42	3.62	0.48	6.57
Born in Uppsala	0.69		2.20	1.04	0.57	2.24	6.05
Born elsewhere	6.68		1.70	1.29	0.78	0.59	7.67
Sibling in area of origin	82.99		1.26	0.91	0.52	0.43	5.00
One parent not in origin: both in origin	89.52		1.35	1.00	0.59	0.48	5.07
In Stockholm	1.08		5.75	0.69	0.57	0.69	5.42
In Gothenburg	0.48		1.11	5.26	0.53	0.29	5.50
In Malmö/Lund	0.19		1.36	1.15	4.12	0.54	5.02
In Uppsala	0.13		1.55	0.42	0.36	5.01	5.66

Elsewhere	8.61		1.37	0.80	0.42	0.35	7.61
Parents' migration history: both born in origin	46.68		1.09	0.75	0.41	0.36	4.76
At least one born in Stockholm	4.88		2.83	0.98	0.65	0.75	6.12
At least one born in Gothenburg	5.24		0.95	2.51	0.59	0.33	5.28
At least one born in Malmö/Lund	2.79		1.26	1.23	2.10	0.47	5.56
At least one born in Uppsala	1.63		2.08	0.99	0.66	1.35	5.90
None born in 4 cities, one elsewhere	28.54		1.60	1.07	0.65	0.60	5.88
None born in 4 cities, one abroad	10.25		1.69	1.06	0.65	0.46	5.61
Parents' highest level of education: primary	6.19		0.73	0.50	0.23	0.12	3.47
Secondary	55.23		0.96	0.67	0.32	0.24	4.66
Post-secondary	38.58		2.12	1.54	0.99	0.87	6.51
Parents' income (SEK 100,000s)	*4.98*	*4.94*					
Parents not together	43.64		1.38	0.84	0.45	0.37	5.23
Distance to Stockholm (100 km)	*3.10*	*1.65*					
Distance to Gothenburg (100 km)	*3.51*	*2.59*					
Distance to Malmö/Lund (100 km)	*4.79*	*3.01*					
Distance to Uppsala (100 km)	*3.04*	*1.58*					
Population size (100,000s)	*1.44*	*0.70*					
Higher education in region (ref. no)	25.65		1.44	0.90	0.47	0.53	7.30
College	42.00		1.27	1.21	0.73	0.43	4.39
University	32.35		1.52	0.80	0.47	0.49	4.89
Unemployment rate	*10.69*	*3.44*					
Year: 2007	15.63		1.41	1.09	0.61	0.51	5.42
2008	16.11		1.46	1.01	0.63	0.49	5.42
2009	16.55		1.41	1.04	0.60	0.47	5.43
2010	16.97		1.39	0.98	0.57	0.46	5.29
2011	17.27		1.36	0.93	0.55	0.49	5.11
2012	17.48		1.34	0.94	0.52	0.44	5.15

Table A3: Average marginal effects (predicted probabilities) from multinomial logistic regression of moving (ref: stayed in region of origin), men

	Stockholm		Gothenburg		Malmö/Lund		Uppsala		Elsewhere	
	b	std error	b	std error	b	std error	b	std error	b	std error
Siblings in Stockholm (ref. no)										
one	0.012	0.001	0.001	0.000	0.000	0.000	0.001	0.000	0.000	0.001
more than one	0.016	0.002	0.000	0.001	0.001	0.001	0.001	0.001	0.000	0.002
Siblings in Gothenburg (ref. no)										
one	0.002	0.000	0.011	0.001	0.000	0.000	0.000	0.000	0.000	0.001
more than one	0.001	0.002	0.014	0.003	0.000	0.001	0.000	0.001	-0.004	0.002
Siblings in Malmö/Lund (ref. no)										
one	0.001	0.001	0.001	0.000	0.007	0.001	0.001	0.000	0.003	0.001
more than one	0.003	0.002	0.001	0.001	0.012	0.002	0.000	0.001	0.008	0.003
Siblings in Uppsala (ref. no)										
one	0.001	0.001	0.001	0.001	0.002	0.000	0.009	0.001	0.002	0.002
more than one	0.000	0.002	-0.001	0.002	-0.003	0.001	0.013	0.002	0.001	0.004

Note: Odds ratios shown in Table 1a. Results for control variables not shown

Table A4: Average marginal effects (predicted probabilities) from multinomial logistic regression of moving (ref: stayed in region of origin), women

	Stockholm		Gothenburg		Malmö/Lund		Uppsala		Elsewhere	
	b	std error	b	std error	b	std error	b	std error	b	std error
Siblings in Stockholm (ref. no)										
one	0.016	0.001	0.000	0.000	0.001	0.000	0.001	0.000	-0.002	0.001
more than one	0.017	0.002	-0.001	0.001	0.000	0.001	0.001	0.001	-0.002	0.002
Siblings in Gothenburg (ref. no)										
one	0.002	0.001	0.016	0.002	0.000	0.000	0.000	0.000	0.001	0.001
more than one	0.004	0.002	0.020	0.002	0.002	0.001	0.001	0.001	-0.002	0.003
Siblings in Malmö/Lund (ref. no)										
one	0.002	0.001	0.003	0.001	0.012	0.002	0.001	0.000	0.001	0.001
more than one	0.001	0.002	0.003	0.002	0.017	0.006	0.003	0.002	-0.006	0.004
Siblings in Uppsala (ref. no)										
one	0.001	0.001	0.001	0.001	0.001	0.000	0.012	0.001	-0.001	0.002
more than one	0.003	0.003	-0.005	0.002	0.005	0.003	0.013	0.003	0.001	0.004

Note: Odds ratios shown in Table 1b. Results for control variables not shown
